# An Extensive Survey of Vertebrate-specific, Nonvisual Opsins Identifies a Novel Subfamily, Q113-Bistable Opsin

**DOI:** 10.1093/gbe/evaf032

**Published:** 2025-03-01

**Authors:** Fuki Gyoja, Keita Sato, Takahiro Yamashita, Takehiro G Kusakabe

**Affiliations:** Department of Biology, Faculty of Science and Engineering, Konan University, Kobe 658-8501, Japan; Department of Biology, Institute for Integrative Neurobiology, Graduate School of Natural Science, Konan University, Kobe 658-8501, Japan; Department of Biophysics, Graduate School of Science, Kyoto University, Kyoto 606-8502, Japan; Department of Biophysics, Graduate School of Science, Kyoto University, Kyoto 606-8502, Japan; Department of Biology, Faculty of Science and Engineering, Konan University, Kobe 658-8501, Japan; Department of Biology, Institute for Integrative Neurobiology, Graduate School of Natural Science, Konan University, Kobe 658-8501, Japan

**Keywords:** vertebrate-wide survey, vertebrate genome evolution, nonvisual photo-sensing, novel opsin subfamily

## Abstract

A group of nonvisual opsins specific to vertebrates is essential to understand evolution of lateral eyes, one of the most prominent innovations in this lineage. Nevertheless, our knowledge of their evolutionary history remains limited. To develop an integrated view of their evolution, we surveyed these non-visual opsins (VA opsin, pinopsin, parapinopsin, parietopsin, and parapinopsin-like) in 451 vertebrate genomes. Through extensive manual curation, we completed a high-quality catalog. We could not find them in 202 mammals, supporting previous reports of their loss. VA opsins are highly conserved among nonmammals. In contrast, other opsin subfamilies experienced more dynamic molecular evolution with many secondary losses. In addition, we found a previously unreported opsin subfamily that we named Q113-Bistable (QB) opsin. We found its orthologs only in several lizards and the tuatara. Nevertheless, QB opsin pseudogenes were discovered in diverse taxa, including ray-finned fishes, indicating its ancient origin. QB opsin, parapinopsin, and parietopsin are extremely prone to be lost in the course of evolution, and loss events involving these opsins seem to occur concomitantly. Furthermore, we demonstrated the spectral properties of QB opsin as a UV-sensitive, bistable photo-pigment. This study provides the first integrated view of the entire evolutionary history of this group of opsins.

**Significance**

Opsins are important proteins in animal photo-sensing. In this study, we focused on a group of nonvisual opsins specific to vertebrates. Despite their important position in understanding the acquisition of sophisticated photoreception systems, our knowledge of their evolutionary history has been limited. We searched for them in a wide range of species. We found a novel subfamily, QB opsin, and characterized its spectral properties. We discovered that the common ancestor of ray-finned fishes and tetrapods must have had QB opsin, although among extant species, only several lizards and the tuatara retain it. We also found that while one opsin subfamily is conserved in most species, others have experienced multiple losses with characteristic patterns. This study provides the first evolutionary overview of these nonvisual opsins.

## Introduction

**Fig. 1.**
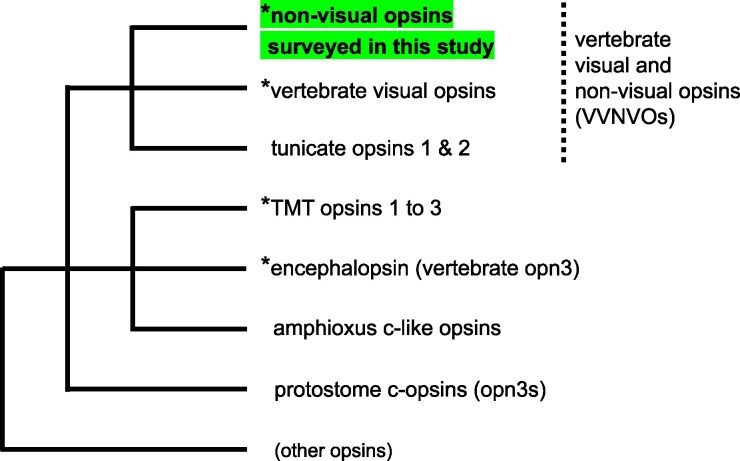
Phylogenetic relationships of VVNVOs and closely related opsins. A tree based on Fischer et al. (2013), Koyanagi et al. (2013), Hara, Takeuchi et al. (2018), Hara, Yamaguchi et al. (2018), and Yamaguchi et al. (2021) is shown. While VVNVOs form a cluster, TMT opsins and encephalopsin (vertebrate opn3) make another cluster. Opsins specific to vertebrates are marked by asterisks. This study focused on nonvisual opsins of VVNVOs. Note that several clades including that of nonvisual opsins of VVNVOs are possibly paraphyletic. Echinoderms also have related opsins, which are omitted in this tree (Ramirez et al. 2016). VVNVOs, vertebrate visual and nonvisual opsins.

Lateral eyes enable color vision with high spatial and temporal resolution and are one of the most notable innovations of vertebrates. How this structure emerged has been a long-standing debate in evolutionary biology. Opsin, a G-protein-coupled receptor protein, is important in metazoan photo-sensing, including that of lateral eyes. Traditionally, opsins have been categorized into several superfamilies (reviewed by Terakita 2005; Shichida and Matsuyama 2009). Among them, vertebrate visual and nonvisual opsins (VVNVOs) are reported only in vertebrates and their closest living relatives, tunicates (for tunicate opsins, see Kusakabe et al. 2001; Kojima et al. 2017). They are not found in the amphioxus genome (Holland et al. 2008). Relationships between this superfamily and closely related opsins, including encephalopsin (vertebrate opn3) and teleost multiple tissue (TMT) opsins, which are also vertebrate-specific, are shown in Fig. 1. In vertebrates, this superfamily was thought to comprise five visual and five nonvisual subfamilies. Visual opsins, RH1, RH2, LWS, SWS1, and SWS2, function mainly in visual perception by rod or cone photoreceptors in the lateral-eye retina. These opsin proteins have several derived molecular properties, including the E113 counterion (Sakmar et al. 1989; Zhukovsky and Oprian 1989; Nathans 1990), a monostable nature, and highly efficient G protein activation ability (Robinson et al. 1992; Jager et al. 1994; Farrens et al. 1996). These molecular properties are likely adaptations to visual photoreception (reviewed by Terakita 2005; Shichida and Matsuyama 2009). In contrast, nonvisual opsins, vertebrate ancient (VA) opsin (Soni and Foster 1997), pinopsin (Okano et al. 1994; Max et al. 1995), parapinopsin (Blackshaw and Snyder 1997), parietopsin (Su et al. 2006), and parapinopsin-like (Kawano-Yamashita et al. 2020) generally function in extraocular photoreceptors, such as those of the pineal gland and related organs. Extraocular photoreceptors are involved in biological functions such as entrainment of the circadian clock, light-dependent body-color change, or control of body temperature. While these nonvisual opsins share some derived characters with visual opsins, they also retain some ancestral characters. For example, parapinopsin forms an ancestral-type bistable photo-pigment (Koyanagi et al. 2004). Nonvisual opsins potentially provide important cues into how visual perception utilizing opsins with unique molecular properties evolved from an ancient vertebrate photoreception system.

Despite their pivotal position in evolutionary studies of vertebrates, our knowledge of the phylogenetic distribution and evolutionary history of these nonvisual opsins remains limited. This limitation can be attributed in part to difficulties in evaluating effects of evolutionary events such as gene duplication, retention, and/or loss of nonvisual opsins on cells, organs, or individuals.

Recent comparative genomics have revealed that genomic contexts affect evolutionary gene retention/loss (Session et al. 2016; Hara, Takeuchi et al. 2018; Parey et al. 2022; Hara and Kuraku 2023). For example, Hara and Kuraku (2023) showed that “elusive” genes encoded in genomic regions with unique characters, such as high repeat element density and high GC content, are much more prone to being lost than their paralogs encoded in genomic regions without those attributes. Biased gene retention/loss has also been reported in genomes that experienced allopolyploidization (Session et al. 2016) or autopolyploidization (Parey et al. 2022). This point of view in combination with classical experimental approaches may allow more integrated insights into the molecular evolution of opsins.

In this study, we extensively surveyed the five nonvisual opsin families in 451 vertebrate genomes. We found that VA opsin orthologs are highly conserved among nonmammalian vertebrates, with only a few secondary losses. Notably, paralogs of this opsin produced by lineage-specific whole genome duplications (WGDs) are also highly conserved, suggesting a tendency for strong retention in genomes regardless of copy number. In contrast, members of the other four opsin subfamilies are much more prone to being lost. In addition, during our survey, we found an opsin subfamily in several lizard species that we named Q113-Bistable (QB) opsin. To our knowledge, this is the first report of this subfamily. We documented its UV-sensitive, bistable spectral properties. Furthermore, we found pseudogenes of this opsin in several taxa, including turtles, coelacanths, sturgeons, and gar. This indicates that the common ancestor of extant Teleostomi already had this opsin. It also suggests that this opsin has been extremely prone to loss during vertebrate evolution. QB opsin, parapinopsin, and parietopsin tend to be lost together. Based on these findings, the evolution of vertebrate visual and nonvisual opsins will be discussed.

## Results

**Fig. 2.**
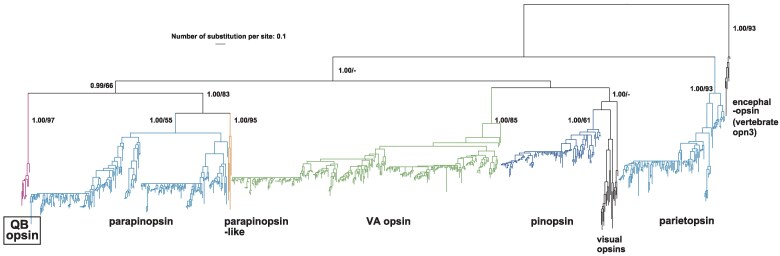
Seven hundred and forty-one nonvisual opsins of VVNVO retrieved in this study were assigned to one of the previously known five subfamilies or the QB opsin subfamily. This tree was generated using the BI method, based on the alignment of G-protein-coupled receptor domains. The ML method yielded a similar result. Posterior probabilities of BI and bootstrap values of ML are shown in this order at major nodes. Visual opsins and encephalopsin (vertebrate opn3) were added to our data set, and the latter was used as an outgroup (supplementary table S4, Supplementary Material online for GenBank/DDBJ/EMBL accession IDs).

To retrieve opsin genes from a wide range of vertebrates, we performed BLAST searches from a refseq database of 202 mammalian and 249 nonmammalian species using VA opsin, pinopsin, parapinopsin, and parietopsin as queries (Materials and Methods). Molecular phylogenetic trees generated with the Neighbor-Joining method (NJ), using a limited number of species, were referenced to annotate these opsin genes. We retrieved opsin genes that belong to the VVNVO superfamily, but do not belong to any previously known visual opsin subfamilies. Hereafter, we refer to them as “nonvisual opsins of VVNVO.” For high-quality cataloging, we performed TBLASTN searches followed by extensive manual gene predictions to retrieve their putative full-length ORFs when automated prediction failed or seemed imprecise. As a result, we retrieved 752 nonvisual opsin genes of VVNVO. Thirty-four were newly predicted or modified. Then, we generated a Bayesian Inference (BI) molecular phylogenetic tree using nearly all of them (Fig. 2). The maximum-likelihood (ML) method also yielded a similar result. Although the limitation of molecular phylogenetic analyses using this number of sequences when the number of informative sites in the alignment is around 250 or fewer should be considered, the resultant tree was largely consistent with previous studies, with one additional clade, the QB opsin subfamily. Repertoires of all species we surveyed are presented in supplementary table S1, Supplementary Material online. GenBank/DDBJ/EMBL accession IDs are provided in supplementary table S2, Supplementary Material online, including those of manually predicted or modified sequences. Molecular phylogenetic trees and alignments are available on FigShare (doi: https://doi.org/10.6084/m9.figshare.27823461.).

Foster and Bellingham (2004) suggested that mammals lack pinopsin. Perry et al. (2018) showed that some mammals, including the Monotremata and Marsupialia, lack VA opsin, pinopsin, parapinopsin, and parietopsin. Even though vertebrate genomic information has expanded greatly in recent years, we could not find any nonvisual opsins of VVNVO in 202 mammals, including two monotremes and eight marsupials (supplementary table S3, Supplementary Material online). Evolutionary events such as secondary losses and gene duplications of nonvisual opsins of VVNVO of nonmammalian species are discussed below.

### VA Opsin

Vertebrate Ancient (VA) opsin was first isolated from the eyes of the Atlantic salmon (Soni and Foster 1997). Yokoyama and Zhang (1997) reported its ortholog from a sea lamprey, suggesting that the common ancestor of extant vertebrates had this opsin. In teleost fishes, two VA opsin paralogs, VA.a and VA.b, were likely produced by the WGD of ancestral teleost fishes (Kojima et al. 2008). Although snake species surveyed apparently lost other nonvisual opsins of VVNVO, they retain a VA opsin ortholog (Castoe et al. 2013; Perry et al. 2018). Borges et al. (2015) showed that 48 bird species surveyed probably retain VA opsin ortholog, although some of them may be pseudogenes.

**Fig. 3.**
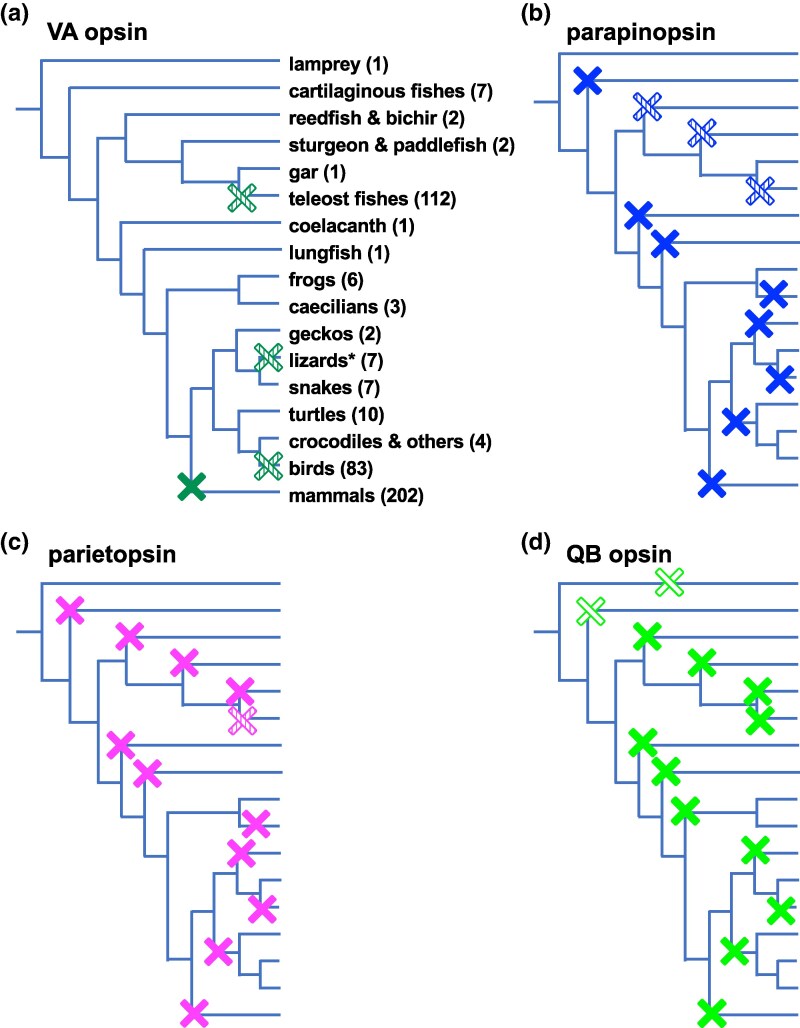
Deduced loss events of a) VA opsin, b) parapinopsin, c) parietopsin, and d) QB opsin are shown on phylogenetic trees. While losses of VA opsin are rare, other opsins are more prone to being lost. Loss events of parapinopsin, parietopsin, and QB opsin seemingly occur concomitantly. Filled Xs indicate that all surveyed species of this clade have lost the specified subfamily. Hatched Xs show that a subset of species of this clade have lost it. Assuming that the common ancestor of extant vertebrates already had QB opsin, deduced losses are indicated by open Xs. Numbers of species surveyed are shown in parentheses. Taxonomic labels are omitted in b to d. Deduced losses in teleost fishes are shown in supplementary fig. S2, Supplementary Material online. Note that for convenience, deduced losses of QB opsin in the ray-finned fish clade are not parsimoniously shown. The “lizard” clade is paraphyletic (shown by an asterisk).

In this study, we retrieved 291 VA opsin genes from 246 nonmammalian species (Fig. 2, supplementary table S1, Supplementary Material online). This opsin is highly conserved among nonmammals, even when other types of nonvisual opsins of VVNVO have been lost. Among nonmammals, we were unable to find VA opsin, including its pseudogenes, in the genome of the swamp eel *Monopterus albus* (supplementary table S1, Supplementary Material online). We also could not find any candidate sequences from Illumina genomic raw reads of this species (SRA accession ID = SRX10193325). VA opsins have become pseudogenes in the Komodo dragon (*Varanus komodoensis*) and the white-throated tinamou (*Tinamus guttatus*) (supplementary fig. S1, Supplementary Material online). Deduced positions of loss events of VA opsin are shown in Fig. 3a and supplementary fig. S2a and b, Supplementary Material online.

Our survey revealed that most euteleosts have a single copy of VA opsin (supplementary table S1, Supplementary Material online). In contrast, outside the euteleost clade, most species have two paralogs, VA.a and b (supplementary table S1, Supplementary Material online). Euteleost VA opsin genes form a single clade with zebrafish VA.a in both ML and BI (supplementary fig. S3a, Supplementary Material online). Microsyntenic analyses also support this orthology (supplementary fig. S3b and c, Supplementary Material online). We propose that euteleost fishes have lost VA.b (supplementary fig. S2a and b, Supplementary Material online).

Most teleost fishes have VA.a orthologs in their genomes, and these genes seem functional. Besides the swamp eel, the only exceptions we could find are two species that belong to the suborder Clupeoidei. These herring species have only VA.b orthologs in their genomes (supplementary table S1, Supplementary Material online; supplementary fig. S2a and b, Supplementary Material online). Furthermore, when parsimoniously deduced, VA.b seems to have been lost only once at the basal position of the Euteleostei (supplementary fig. S2b, Supplementary Material online). In summary, the loss of VA opsin seems quite rare, even if there are two or more paralogs in the genome.

In further support of this speculation, all species that have experienced clade-specific WGD also retain duplicated VA opsin paralogs (supplementary table S1, Supplementary Material online). In particular, all three carp species and their relatives (Cypriniformes) have two VA.a paralogs and two VA.b paralogs. We could not find any unnatural features in their predicted amino acid sequences, such as large deletion(s). We speculate that these four paralogs are functional. One may imagine that retention of many VA opsin paralogs may be adaptive. Nevertheless, we could not find any tandem duplication events of VA opsins throughout our survey (supplementary table S1, Supplementary Material online). We suggest that the high retention propensity of this opsin may not necessarily be attributable to functional necessity alone.

### Pinopsin

The first report of pinopsin was from chickens (Okano et al. 1994). Hara, Yamaguchi et al. (2018) reported its ortholog in genomes of several shark species, suggesting that the common ancestor of gnathostomes had this opsin. Pinopsin is thought to have been lost among teleost fishes (Foster and Bellingham 2004), while it is retained in their close relatives, the spotted gar, the gray bichir, and the Siberian sturgeon (Sato et al. 2018). This opsin has been lost in several snake species (Castoe et al. 2013; Perry et al. 2018). Emerling (2017) showed that pinopsin genes have become pseudogenes in four crocodile species. Borges et al. (2015) reported pinopsin loss events in six of 48 bird species.

We found 110 pinopsin genes in 108 species (Fig. 2, supplementary table S1, Supplementary Material online). Deduced positions of pinopsin loss events are shown in supplementary figs. S2c and S4a, Supplementary Material online. Pinopsin seems more prone to loss than VA opsin. We could not find this opsin in the sea lamprey (*Petromyzon marinus*) genome. We also failed to find pinopsin in genomes of the Arctic lamprey (*Lethenteron camtschaticum*) (Nakatani et al. 2021), the pouched lamprey (*Geotria australis*) (GenBank accession ID: GCA_036362915), the brown hagfish (*Eptatretus atami*) (Marlétaz et al. 2024), or the inshore hagfish (*E. burgeri*) (Yu et al. 2024). We suggest that this subfamily was lost secondarily in the cyclostome clade (supplementary fig. S4a, Supplementary Material online).

We found that in tetrapods, pinopsin has experienced several losses in addition to those already reported. Two of three cecilian (Gymnophiona) species have lost pinopsin (supplementary table S1, Supplementary Material online), as have some birds. First, in the order Galliformes (chicken, pheasant, and others), pinopsins of two grouses have become pseudogenes (supplementary fig. S5, Supplementary Material online). Seven other galliform species, including the turkey, the closest known relative of grouses, have pinopsin orthologs in their genomes (Borges et al. 2015 for chicken and turkey pinopsins; Wang et al. 2013 for galliform phylogeny). Therefore, pseudogenization likely occurred very recently in this clade. Second, we found that all palaeognath species we surveyed have lost functional pinopsin. Extant birds are largely divided into two clades, the Palaeognathae (the tinamous and flightless ratites) and the Neognathae (all other extant birds) (Jarvis et al. 2014 and references therein). Borges et al. (2015) reported that they could not find pinopsin in genomes of two palaeognath species, the common ostrich (*Struthio camelus*) and the white-throated tinamou (*T. guttatus*). In addition, we discovered that in the emu and kiwi genomes, pinopsin has become a pseudogene, whereas we could not find any candidate in the Chilean tinamou genome (supplementary fig. S5, Supplementary Material online). We also found a pinopsin pseudogene in the common ostrich genome (supplementary fig. S5, Supplementary Material online). We could not judge whether the loss of pinopsin occurred once at the base of the Palaeognathae, because many of these pseudogenes are highly fragmented and we could not find any shared mutations.

Recently, we isolated a pinopsin ortholog from the tarpon (*Megalops*), a basal teleost (Fujiyabu et al. 2024). In this study, we could not find pinopsin in genomes of any other teleost fishes (supplementary table S1, Supplementary Material online, supplementary fig. S2c, Supplementary Material online).

### Parapinopsin

This opsin was first isolated from a parapineal organ of the channel catfish (Blackshaw and Snyder 1997). Koyanagi et al. (2004) isolated it from the river lamprey in addition to the clawed frog and the trout, showing that it is of ancient origin. Several snakes, as well as crocodiles and geckos, seem to have lost parapinopsin, while it is retained in some lizards (Castoe et al. 2013; Perry et al. 2018). Emerling (2017) reported the absence of functional parapinopsin in four turtles as well as five birds. Borges et al. (2015) showed its loss in 48 birds. Hara, Yamaguchi et al. (2018) could not find parapinopsin in elasmobranch shark genomes they surveyed. Teleost fishes have two parapinopsin paralogs in their genomes, parapinopsins.a and b. They are thought to have been produced by the WGD of teleost fishes (Koyanagi et al. 2015).

We found 215 parapinopsin genes in 120 species (Fig. 2, supplementary table S1, Supplementary Material online). We could not find them in the genomes of three cecilians, the coelacanth, or the lungfish (supplementary table S1, Supplementary Material online). Through extensive searches, we found parapinopsin orthologs in genomes of the sea lamprey, most teleost fishes, frogs, and most lizards, largely consistent with previous studies. Despite our thorough survey, we could not find parapinopsin in refseq_genomes of either cartilaginous fishes, birds, turtles, snakes, geckos, or crocodilians, also largely consistent with previous studies (supplementary table S1, Supplementary Material online). Deduced positions of loss events of parapinopsin are shown in Fig. 3b and supplementary figs. S2d and e, Supplementary Material online.

Although many teleost fishes we surveyed have both parapinopsins.a and b in their genomes, several species seem to have lost either or both of them secondarily (supplementary table S1, Supplementary Material online, supplementary fig. S2d and e, Supplementary Material online). For example, in the order Cyprinodontiformes (killifishes and others), loss events of parapinopsin.a seem to have occurred at least twice, once in the Aplocheiloidei clade and once in the Poeciliidae clade (supplementary fig. S2d, Supplementary Material online). Loss of parapinopsin.b seems to have occurred in the Rivulidae clade, resulting in the complete loss of parapinopsin in this clade (supplementary fig. S2e, Supplementary Material online).

Many species that experienced recent WGD, namely those belonging to Acipenseriformes (sturgeons and paddlefishes), carp and salmon species, and the African clawed frog have lost one or more paralogs of parapinopsin (supplementary table S1, Supplementary Material online), contrasting with VA opsin.

### Parietopsin

This opsin was first isolated from the parietal eye of the side-blotched lizard (Su et al. 2006). The authors also found its orthologs *in silico* in the clawed frog, the zebrafish, and fugu (Su et al. 2006). Wada et al. (2021) isolated its ortholog from the river lamprey, indicating that the common ancestor of extant vertebrates already had parietopsin. Emerling (2017) reported the absence of functional parietopsin in four turtles as well as six birds. Castoe et al. (2013) and Perry et al. (2018) reported the loss of parietopsin in several snakes. Borges et al. (2015) could not find parietopsin in any of 48 bird species they surveyed. Sharks also seem to have lost parietopsin (Hara, Yamaguchi et al. 2018).

We found 115 parietopsin genes in 112 species (Fig. 2, supplementary tables S1 and S2, Supplementary Material online), but we failed to find parietopsin in genomes of three cecilian species, the coelacanth, or the lungfish (supplementary table S1, Supplementary Material online). Despite extensive searches, we could not find parietopsin in cartilaginous fishes or birds, largely consistent with previous studies (supplementary table S1, Supplementary Material online). Turtles, snakes, and crocodilians likely have also lost this opsin, consistent with previous studies (supplementary table S1, Supplementary Material online). On the other hand, we found that most lizards retain parietopsin (supplementary table S1, Supplementary Material online). As shown in Fig. 3c, this opsin also has experienced many secondary losses. This loss pattern seems to be quite similar to that of parapinopsin (Fig. 3b). Losses of parietopsin in teleost fishes also likely occurred together with losses of parapinopsin.a and/or b (supplementary fig. S2d and e, f, Supplementary Material online).

In the African clawed frog, the S paralog of parietopsin has become a pseudogene (Gene ID = LOC108713178). After WGD in the Cypriniformes (carp and their relatives) and Salmoniformes (salmon and trout), nearly all species retain only one paralog (supplementary table S1, Supplementary Material online).

We found a possible duplication event in one small clade. Two teleost fishes, the red-bellied piranha (*Pygocentrus nattereri*) and the tambaqui (*Colossoma macropomum*), both belonging to the order Characiformes, have two parietopsin paralogs (supplementary table S1, Supplementary Material online). This seems to be an interesting exception.

### Parapinopsin-like

Recently Kawano-Yamashita et al. (2020) reported that there is another nonvisual opsin subfamily of VVNVO in vertebrates. This subfamily tends to be clustered with parapinopsin, but forms a distinct cluster in molecular phylogenetic analyses (Lamb et al. 2016; Kawano-Yamashita et al. 2020). At present, its orthologs are reported only in lampreys and the elephant shark (Kawano-Yamashita et al. 2020). We failed to find its orthologs in any phylogenetically distant species other than those already reported by Kawano-Yamashita et al. (2020). Our molecular phylogenetic analysis showed that extant lampreys have three paralogs of this subfamily (supplementary fig. S6, Supplementary Material online; also see Yamaguchi et al. 2021). In the sea lamprey, these three paralogs are assigned on chromosomes 14, 42, and 48 (supplementary table S2, Supplementary Material online). Since most parts of these three chromosomes are proposed to have originated from a single, ancient chromosome (Marlétaz et al. 2024), we speculate that these three paralogs arose by an ancient genome triplication in this lineage (Nakatani et al. 2021; Marlétaz et al. 2024; Yu et al. 2024). Deduced positions of loss events in parapinopsin-like are shown in supplementary fig. S4b, Supplementary Material online.

### QB Opsin

During this survey, we noticed that several lizard opsins do not belong in any known subfamilies in molecular phylogenetic trees (Fig. 2, supplementary fig. S6, Supplementary Material online). They form a statistically significant single clade (posterior probability of BI = 1.00, bootstrap value of ML = 100% in supplementary fig. S6, Supplementary Material online). We also found its ortholog in the tuatara (*Sphenodon punctatus*) genome (Gemmell et al. 2020) (supplementary fig. S6, Supplementary Material online). This opsin subfamily forms a single clade with parapinopsin and parapinopsin-like, but placed outside them (Fig. 2, supplementary fig. S6, Supplementary Material online). Based on its position in dendrograms, we speculate that these genes comprise a novel nonvisual opsin subfamily of VVNVO. We named it Q113-Bistable (QB) opsin.

**Fig. 4.**
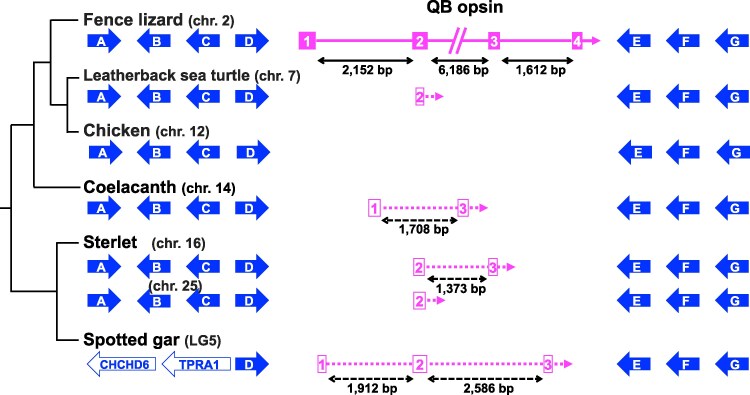
Microsynteny around the lizard QB opsin locus is highly conserved in phylogenetically distant species. Pseudogenized sequence segments of QB opsin corresponding to one to three exon(s) were found from the leatherback sea turtle (*Dermochelys coriacea*), the coelacanth (*Latimeria chalumnae*), the sterlet (*Acipenser ruthenus*), and the spotted gar (*Lepisosteus oculatus*). Protein-coding genes are shown. Distances between these segments are based on the TBLASTN search results (supplementary fig. S9, Supplementary Material online). A = NT5DC2, B = STAB1, C = NISCH, D = TNNC1, E = RPL29, F = DUSP7, and G = POC1A. GenBank/DDBJ/EMBL accession IDs of A to G of these species are shown in supplementary table S5, Supplementary Material online. Orthology of NT5DC2 genes between species was confirmed by molecular phylogenetic analyses (supplementary fig. S7a, Supplementary Material online). TNNC1 genes used were confirmed as TNNC1, not 2 (supplementary fig. S7b, Supplementary Material online).

Molecular phylogenetic analyses imply that the origin of the QB opsin subfamily may trace back to the common ancestor of extant vertebrates (Fig. 2, supplementary fig. S6, Supplementary Material online). To assess its evolutionary origin further, we surveyed microsyntenic conservation around its locus. Despite the absence of its ortholog, the corresponding microsynteny block was conserved in the chicken, a turtle, the coelacanth, the sterlet, and the gar (Fig. 4). This is also true of parapinopsin (supplementary fig. S8, Supplementary Material online). These data suggest that both of these microsynteny blocks represent the ancestral state of extant Teleostomi, and that they have not experienced any breakage due to genomic rearrangements such as chromosomal inversion or translocation in these lineages. Furthermore, coding regions of QB opsins have a four-exon/three-intron structure, like those of parapinopsins. Based on the foregoing evidence, we propose that QB opsin is not likely a recently emerged parapinopsin paralog produced either by a tandem duplication or a retrotransposition event.

### Pseudogenes of QB Opsin

To further investigate the evolutionary origin of QB opsin, we searched for its pseudogenes. We found candidates in a turtle, the coelacanth, the sterlet, and the gar (Fig. 4), as well as the alligator and the Japanese gecko (supplementary fig. S9, Supplementary Material online). To assess whether they are bona fide QB opsin pseudogenes, we performed reciprocal BLAST searches and molecular phylogenetic analyses. Using predicted amino acid sequences of the corresponding region of each exon, we performed BLASTP searches against the refseq_protein database of the fence lizard (*Sceloporus undulatus*). For all queries, the best hit protein was a QB opsin (supplementary table S6, Supplementary Material online). Furthermore, those pseudogenized nucleotide sequences make a statistically significant single clade with fence lizard and tuatara QB opsins in molecular phylogenetic trees (supplementary fig. S10, Supplementary Material online, bootstrap values of ML = 100% for regions corresponding to the second and third exons of QB opsin respectively). We conclude that they are bona fide QB opsin pseudogenes.

Deduced positions of loss events involving QB opsin are shown in Fig. 3d. Since some ray-finned fishes have pseudogenes, the common ancestor of extant Teleostomi should have had this subfamily. We also noted that assuming as few as three additional secondary loss events to those of parietopsin would reproduce the same distribution pattern as QB opsin (Fig. 3c and d).

Despite vigorous searches, we could not find any pseudogene candidates in genomes of five cyclostome species, the Arctic lamprey (*L. camtschaticum*) (Nakatani et al. 2021), the pouched lamprey (*G. australis*) (GCA_036362915), the brown hagfish (*E. atami*) (Marlétaz et al. 2024), or the inshore hagfish (*E. burgeri*) (Yu et al. 2024). We also could not find any in nine species of Chondrichthyes (supplementary table S1, Supplementary Material online), the zebra shark (*Stegostoma tigrinum*) (GCF_030684315) or the spotted ratfish (*Hydrolagus colliei*) (GCA_035084275).

### Spectral Properties of QB Opsin

**Fig. 5.**
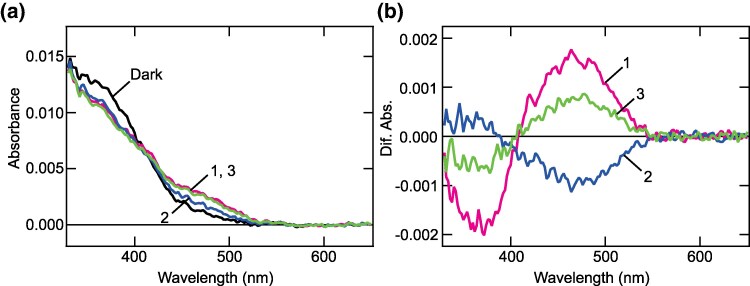
Spectral properties of green anole QB opsin. a) Absorption spectra of green anole QB opsin purified after reconstitution with 11-*cis* retinal. Spectra were recorded in the dark, after UV light (360 nm) irradiation (curve 1), subsequent yellow light (>500 nm) irradiation (curve 2) and UV light re-irradiation (curve 3). b) Spectral changes induced by UV light irradiation (curve 1), subsequent yellow light irradiation (curve 2) and UV light re-irradiation (curve 3). Difference spectra were calculated based on the spectra shown in a).

Spectral properties of at least one member of each previously known nonvisual opsin subfamily of VVNVO have been reported (Okano et al. 1994; Kojima et al. 2000; Koyanagi et al. 2004; Su et al. 2006; Sato et al. 2011; Sakai et al. 2012; Kawano-Yamashita et al. 2020). Therefore, that of QB opsin is the last gap remaining. Accordingly, we characterized spectral properties of green anole QB opsin. We expressed the recombinant protein in HEK293T cells incubated with 11-*cis* retinal and obtained purified photosensitive pigments. Spectroscopic analyses showed that its absorption spectrum has a main peak in the UV region, around 370 nm (black curve in Fig. 5a). Irradiation with UV light induced a decrease in absorption around 370 nm and an increase around 470 nm (magenta curves in Fig. 5a and b). Subsequent yellow light irradiation induced a decrease in absorption around 470 nm and an increase around 370 nm (blue curves in Fig. 5a and b). Furthermore, the third irradiation with UV light repeated the spectral change, with a decrease around 370 and an increase around 470 nm, similar to the first UV irradiation (green curves in Fig. 5a and b). These results indicate that this opsin has UV-sensitive and bistable properties, like lamprey parapinopsin (Koyanagi et al. 2004).

## Discussion

In this study, we surveyed nonvisual opsins of VVNVO in a wide range of vertebrate genomes and created a high-quality catalog of them. While VA opsin is retained in most nonmammal species, other opsin subfamilies experienced more dynamic molecular evolution. In addition, we report a sixth subfamily, QB opsin. We characterized its spectral properties showing that it is a UV-sensitive, bistable photo-pigment. Our survey did not identify any other novel opsin subfamilies. We believe that the QB opsin subfamily completes the inventory of ancient opsin subfamilies of this type in extant vertebrates.

### A Novel Opsin Subfamily, QB Opsin

Here, we report a novel opsin subfamily, QB opsin. We named it after its bistable spectral property as well as an intriguing feature of its predicted amino acid sequence. Generally, opsin photo-sensitivity is achieved by a chromophore, such as 11-*cis* retinal. The chromophore binds to a specific lysine residue of these proteins via a protonated Schiff-base linkage to be isomerized upon absorption of a photon (reviewed by Terakita 2005; Shichida and Matsuyama 2009). Visible light-sensing opsins have the protonated Schiff-base, which is stabilized by a specific negatively charged amino acid as a counterion. Visual opsins, pinopsin, and VA opsin have a conserved glutamic acid at position 113 (E113) and are thought to utilize it as a counterion (Sakmar et al. 1989; Zhukovsky and Oprian 1989; Nathans 1990) (based on bovine rhodopsin numbering). Acquisition of the E113 counterion in the course of VVNVO evolution is thought to have had a great impact on molecular properties of these opsins (reviewed by Shichida and Matsuyama 2009). Parapinopsin and parapinopsin-like also have glutamic acid or aspartic acid at position 113, although lamprey and tetrapod parapinopsin and teleost parapinopsin.a are UV-sensitive opsins with a deprotonated Schiff-base and do not need a counterion. In contrast, QB opsins have glutamine instead of glutamic acid at position 113 (Q113; supplementary fig. S6, Supplementary Material online) as in the case of parietopsin (Su et al. 2006; Sakai et al. 2012). It should be noted that QB opsin and parietopsin have conserved E181, an ancestral counterion position (Terakita et al. 2004).

Considering that VVNVOs have been extensively studied by both experimental biologists and genomic scientists, it was unexpected that a novel opsin subfamily remained to be reported. As far as we have been able to determine, the first appearance of a QB opsin ortholog in a public database is that of the green anole, *Anolis carolinensis*. Its initial genome assembly released in 2009 (Alföldi et al. 2011) contains the QB opsin locus (scaffold_44 of anoCar1, 1169612 to 1177220). Alföldi et al. (2011) surveyed opsin subfamilies that are retained in the lizard, but have been lost in mammals. However, QB opsin is not on their list. Although Perry et al. (2018) surveyed opsin subfamilies from several lizards, snakes, and geckos, we could not find QB opsin in their list either. Hara, Takeuchi et al. (2018) surveyed opsins in genomes of the Madagascar ground gecko and its relatives. A molecular phylogenetic tree in their supplementary data, Supplementary Material online contains the anole lizard QB opsin (ENSACAP00000006735), although they did not mention it. The reason that QB opsin was not mentioned in these studies is not clear. However, since it is not placed with any known VVNVO subfamilies and since only a small number of extant squamate species retain the ortholog, it may have been difficult to conclude that QB opsin comprises a novel opsin subfamily. This study, together with our previous work (Gyoja et al. 2012), illustrates how unbiased searches for groups of gene families sometimes results in identification of a novel member.

### Evolutionary History of Opsin Subfamilies Involving WGDs

The VVNVO superfamily is thought to have emerged in the common ancestor of vertebrates and their sister clade, tunicates. In vertebrates, this superfamily was thought to comprise five visual and five nonvisual opsin subfamilies. In this study, we added a sixth nonvisual member, QB opsin. How this modern-day repertoire emerged has been a matter of long-standing debate (Larhammar et al. 2009; Lagman et al. 2013; Lamb and Hunt 2017; Hofmann and Lamb 2023). One major factor in this complicated situation may be the difficulty in determining whether a given duplication event arose from a local duplication or from WGD. Recent advances in genomic studies in cyclostomes permitted further insights into these events. After sharing 1R with gnathostomes, cyclostomes independently experienced a genome triplication (2R_CY_) (Nakatani et al. 2021; Marlétaz et al. 2024; Yu et al. 2024). We present a scenario illustrating how QB opsin, parapinopsin, and parapinopsin-like subfamilies emerged in the course of evolution (supplementary fig. S11, Supplementary Material online).

We propose that parapinopsin and parapinopsin-like emerged by 1R because (1) these two subfamilies form a statistically significant single clade (posterior probability of BI = 1.00, bootstrap value of ML = 84% in supplementary fig. S6, Supplementary Material online), (2) lampreys have three parapinopsin-like paralogs in their genomes (also see Yamaguchi et al. 2021), and these are located on paralogous chromosomes in the sea lamprey; therefore, they likely resulted from 2R_CY_ (supplementary fig. S11, Supplementary Material online), and (3) the elephant shark has one parapinopsin-like ortholog, indicating that the common ancestor of cyclostome and gnathostome had this subfamily (Kawano-Yamashita et al. 2020). QB opsin is placed outside of parapinopsin and parapinopsin-like (Fig. 2, supplementary fig. S6, Supplementary Material online). Furthermore, microsynteny blocks around QB opsin and parapinopsin loci as well as their corresponding microsynteny blocks are located on the same chromosomes in all six species we surveyed (Fig. 4, supplementary fig. S8, Supplementary Material online). Notably, in the chicken genome, both are located on chr. 12, although both QB opsin and parapinopsin orthologs are missing in this species (Fig. 4, supplementary fig. S8, Supplementary Material online). Since Huang et al. (2023) reported that chicken chr. 12 experienced no major fusion events in the course of vertebrate evolution, we suggest that QB opsin and parapinopsin were located on the same ancestral vertebrate chromosome, and that parapinopsin and QB opsin were produced by a local tandem duplication preceding WGDs (supplementary fig. S11, Supplementary Material online).

Other VVNVOs of vertebrates must have existed in ancestral vertebrates before 2R (supplementary fig. S11, Supplementary Material online), as Lamb and Hunt (2017) and Hofmann and Lamb (2023) suggested. Except for three lamprey parapinopsin-likes, vertebrates seem to have lost all but one paralog after 2R (supplementary fig. S11, Supplementary Material online). Detailed characterization of evolutionary events involving ancestral chromosomes, such as tandem duplications and translocations, will be an intriguing research topic in the future. Our high-quality catalog of nonvisual opsins of VVNVO will serve as a valuable foundation for such further inquiries.

### Evolutionary History of Opsin Subfamilies After WGDs

We found that while VA opsin orthologs are highly conserved among nonmammalian vertebrates, this is not the case for other nonvisual opsins of VVNVO. In particular, the parapinopsin, parietopsin, and QB opsin subfamilies experienced far more dynamic molecular evolution with many secondary losses. We could not find any tandem duplication events involving these six nonvisual opsins. This contrasts with the case of cone opsins such as LWS or RH2. They experienced several tandem duplications during evolution (reviewed by Musilova et al. 2021). Nevertheless, we found that paralogs of VA opsin are highly retained in genomes after lineage-specific WGDs. We suggest that this “prone-to-be-retained” property of VA opsin cannot simply be attributed to its functional necessity alone.

This situation differs from cases of other opsin subfamilies. They are much more prone to loss (Fig. 3, supplementary figs. S2 and S4, Supplementary Material online). In nearly all lineage-specific WGD cases we surveyed, that is, those in the Acipenseriformes (sturgeons and paddlefish), carp and salmon species, and the African clawed frog, parapinopsin and parietopsin have lost one or more paralogs (supplementary table S1, Supplementary Material online). One possible cause of these differences may be the genomic context in which they are located. Recent studies have shown that often the genomic context strongly influences whether a given gene will be retained or lost (Session et al. 2016; Hara, Takeuchi et al. 2018; Hara and Kuraku 2023). For example, Hara, Takeuchi et al. (2018) focused on “elusive” genes that are conserved in two or more reptilian species and are absent from birds or mammals. They showed that genomic regions in which they are located tend to exhibit high-repeat-element density, high gene density, and high GC content. Among many elusive genes, they characterized parapinopsin and parietopsin, as well as TMT opsins (Hara, Takeuchi et al. 2018). TMT opsins are also specific to vertebrates (Fig. 1). Hara, Yamaguchi et al. (2018) and Yamaguchi et al. (2021) showed that among chondrichthyan species, TMT1 tends to be retained in genomes, while paralogous TMT2 and 3 are more prone to be lost. TMT3 possesses larger *K_A_* (nonsynonymous substitutions per site) values between the Madagascar ground gecko and the green anole than those of its nonelusive paralogs, suggesting that its elusiveness is associated with asymmetric evolutionary rates (Hara, Takeuchi et al. 2018). TMT opsins were not included in the present study because they belong to a separate cluster from VVNVOs in a molecular phylogenetic tree (Fig. 1). However, TMT opsins together with VVNVOs occupy an important position in examining the possibility that genomic contexts affect dynamic and complicated molecular evolution of opsins in vertebrates.

There are at least two notable exceptions to the “paralog-loss tendency” of parapinopsin. The first is parapinopsins.a and b, which likely resulted from the WGD of teleost fishes. The second is parapinopsin and parapinopsin-like, which likely emerged from 1R, as discussed in the previous section. Both cases are characterized by the acquisition of novel spectral sensitivity in one of them after duplication (Koyanagi et al. 2015; Kawano-Yamashita et al. 2020). This may have resulted in the survival of two or more paralogs.

## Conclusions

We surveyed five previously known vertebrate-specific nonvisual opsins in a wide range of species. Through extensive manual curation, we constructed a high-quality catalog. Our study revealed unique aspects regarding propensities for retention or loss of each opsin subfamily. Based on these tendencies, genomic context may have influenced the evolution of these opsins after WGDs. We also report a sixth member, QB opsin. The origin of this opsin subfamily traces back to the common ancestor of extant Teleostomi. We determined the spectral properties of lizard QB opsin as a UV-sensitive, bistable photo-pigment. We believe that the present data on QB opsin complete the inventory of extant subfamilies of nonvisual opsins of VVNVO. This study integrates our knowledge of evolutionary history of these opsins, permitting systematic insights into acquisition of vertebrate photoreception.

## Materials and Methods

### BLAST Searches

BLAST searches were performed against the refseq_protein as well as the refseq_genome database from January 2023 to March 2024. Predicted vertebrate visual and nonvisual opsins were searched using BLASTP searches with evalue <1e^−50^. Using this threshold, we also retrieved encephalopsins and TMT opsins, and they were used as an outgroup. Queries for BLAST searches were as follows: AAA30674 (*Bos taurus* rhodopsin), AAZ79904 (*Uta stansburiana* parietopsin), AAA64223 (*Gallus gallus* pinopsin), AAB84050 (*Ictalurus punctatus* parapinopsin), and AAC60124 (*Salmo salar* VA opsin). If we failed to find nonvisual opsin(s) of VVNVO in a given species, a TBLASTN search against the refseq_genome database was performed. If we judged that the automatic gene prediction had failed or was not precise, manual gene predictions were performed using Genscan (Burge and Karlin 1997), Augustus (Stanke et al. 2008), or exon-intron comparisons with orthologs of other species. If we obtained two or more gene models with high sequence similarities, we surveyed chromosomal data, if available, to judge whether they belong to different loci in the genome (supplementary table S1, Supplementary Material online).

### Molecular Phylogenetic Analyses

Amino acid sequences were aligned using MAFFT (Katoh et al. 2002; version 7.245) or ClustalW (Thompson et al. 1994; version 2.1). Gaps and unaligned regions were removed using gblocks (Talavera and Castresana 2007). Molecular phylogenetic analyses were then performed using BI and the ML method with the LG + G model. BI analyses were performed using MrBayes (Huelsenbeck and Ronquist 2001; version 3.2.3) with the following parameters: ngen = 200,000,000 printfreq = 10,000 samplefreq = 100 nchains = 4 temp = 0.2 checkfreq = 50,000 diagnfreq = 500,000 stopval = 0.01 stoprule = yes. The first 25% of these trees were discarded as “burn-in.” Convergence of each run was assessed by plotting the log-likelihood. ML analyses were performed using RAxML-NG (Kozlov et al. 2019; version 1.0.2) with 500 or 1,000 bootstrap pseudoreplications.

For annotation of individual opsin genes (supplementary tables S1 and S2, Supplementary Material online), local molecular phylogenetic trees generated with the NJ were referenced. BioNJ analyses by SEAVIEW (Galtier et al. 1996; version 4.5.4) were performed with 1,000 bootstrap pseudoreplications.

### Preparation and Spectral Measurements of Recombinant Opsin Protein

The DNA fragment corresponding to the full-length ORF of green anole (*Anolis carolinensis*) QB opsin cDNA was commercially synthesized by Medical & Biological Laboratories Co., Ltd. cDNA of the QB opsin was tagged with the epitope sequence of the anti-bovine rhodopsin monoclonal antibody Rho1D4 (ETSQVAPA) at the C-terminus and was inserted into the mammalian expression vector pCAGGS (Niwa et al. 1991). Plasmid DNA was transfected into HEK293T cells using the calcium phosphate method. One day after transfection, the medium was supplied with 5 μM 11-*cis* retinal, and cells were kept in the dark thereafter. Forty-eight hours after transfection, cells were collected. The following procedures were carried out on ice under dim red light. Opsin protein was extracted with 1% dodecyl maltoside (DM) in buffer A (50 mM HEPES (pH 7.0), 140 mM NaCl and 3 mM MgCl_2_) and was purified using Rho1D4 antibody-conjugated agarose. Purified opsin protein was eluted with buffer A containing 0.02% DM and 0.45 mg/mL synthetic peptide with the Rho1D4 epitope sequence. Absorption spectra were recorded with a Shimadzu UV2400 spectrophotometer. Sample temperature was maintained at 0 ± 0.1 °C with an optical cell holder connected to a Neslab RTE-7 temperature controller. Irradiation of the sample was performed with light through a Y-52 cutoff filter or a UV-D36 glass filter (Toshiba Co., Ltd.) from a 1-kW tungsten halogen lamp (Rikagaku Seiki).

## Supplementary Material

evaf032_Supplementary_Data

## Data Availability

Manually predicted/improved sequences are available on DDBJ at their respective accession numbers (BR002503-BR002538). Alignments for opsins and molecular phylogenetic tree files are available on FigShare (doi: https://doi.org/10.6084/m9.figshare.27823461 and doi: https://doi.org/10.6084/m9.figshare.28300961). Other data supporting this article are available in the article and in its online Supplementary material.
