# Correction to: An Extensive Survey of Vertebrate-specific, Nonvisual Opsins Identifies a Novel Subfamily, Q113-Bistable Opsin

**DOI:** 10.1093/gbe/evaf145

**Published:** 2025-07-31

This is a correction to: Fuki Gyoja, Keita Sato, Takahiro Yamashita, Takehiro G Kusakabe, An Extensive Survey of Vertebrate-specific, Nonvisual Opsins Identifies a Novel Subfamily, Q113-Bistable Opsin, *Genome Biology and Evolution*, Volume 17, Issue 3, March 2025, https://doi.org/10.1093/gbe/evaf032

In the originally-published online version of the manuscript, the second author was marked up erroneously as co-corresponding author. This is now corrected so that only Dr Gyoja is the corresponding author for this paper.

