# Supplementary material for: An Extensive Survey of Vertebrate-specific, Nonvisual Opsins Identifies a Novel Subfamily, Q113-Bistable Opsin
Source: Genome Biol Evol. 2025 Mar 1;17(3):evaf032. doi: 10.1093/gbe/evaf032 (PMC11893379; doi:10.1093/gbe/evaf032)
Supplement: evaf032_Supplementary_Data [file evaf032_supplementary_data.zip › 20250211.Supplementary_Figures.docx]

**Supplementary Figures**

**Supplementary Figure S1**

Structures of VA opsin pseudogenes of the Komodo dragon, *Varanus komodoensis*, and the white-throated tinamou, *Tinamus guttatus*. VA opsins have five exons. The VA opsin locus of the fence lizard is shown for comparison. GenBank/DDBJ/EMBL accession IDs of predicted proteins are provided in Supplementary Table S5. stop = in frame stop codon.

**Supplementary Figure S2**

Deduced loss-events of (A) VA.a, (B) VA.b, (C) pinopsin, (D) parapinopsin.a, (E) parapinopsin.b, and (F) parietopsin are shown on phylogenetic trees of teleost fishes. Filled Xs indicate that all species surveyed of a given clade appear to have lost the subfamily in question. Numbers of species surveyed are shown in parentheses.

**Supplementary Figure S3**

(A) A molecular phylogenetic tree of VA opsins of teleost fishes, using Bayesian Inference (BI). The Maximum Likelihood method (ML) yielded a similar result. Posterior probabilities of BI and bootstrap values of ML are shown in this order at major nodes. VA opsins comprise two large clades. One contains zebrafish VA.a, and the other VA.b. VA.b seems to have been lost at the base of the Euteleostei. This result seems partially inconsistent with a previous study (Kojima et al. 2008), in which it was reported that VA opsin sequences from three euteleosts, salmon (*Salmo salar*), ayu (*Plecoglossus altivelis*), and torafugu (*Takifugu rubripes*), are clustered in VA.b. We also included these sequences in our analysis, and these are clustered in VA.a. Although the reason for this inconsistency is not clear at present, we assume that our results are likely more reliable because (1) our study uses sequence data from a much wider array of taxa, owing to recent advances in genomic information from teleost fishes (2) microsynteny analysis shown in (B) and (C) also supports this orthology. (B) Conservation of microsynteny around VA.a loci of zebrafish, tarpon (*Megalops cyprinoides*), salmon, and torafugu. (C) Conservation of microsynteny around VA.b loci of zebrafish and tarpon. Protein coding genes are shown. GenBank/DDBJ/EMBL accession IDs of predicted proteins are provided in Supplementary Table S5. GABRP/gabrz and adgra1/adgra1a in (B) and (C) show sequence similarity. They may be paralogs that originated from the WGD of teleost fishes. We cannot provide detailed relationships between them due to the low resolution of molecular phylogenetic analyses (data not shown).

Kojima D, Torii M, Fukada Y, Dowling JE. 2008. Differential expression of duplicated VAL-opsin genes in the developing zebrafish. J Neurochem. 104:1364-1371.

**Supplementary Figure S4**

Deduced loss-events of (A) pinopsin, and (B) parapinopsin-like are shown on phylogenetic trees. Filled Xs indicate that all surveyed species of this clade have lost the specified subfamily. Hatched Xs show that a subset of species of this clade have lost it. Assuming that the common ancestor of extant vertebrates already had pinopsin, deduced timing of the loss is indicated by an open X. Numbers of species surveyed are shown in parentheses. Deduced losses in teleost fishes are shown in Supplementary Fig. S2. The "lizard" clade is paraphyletic (shown by an asterisk).

**Supplementary Figure S5**

Structures of pinopsin pseudogenes in birds. Pinopsins have five exons. The chicken pinopsin locus is shown for comparison. Pinopsins of two grouses, *Centrocercus urophasianus* (Greater sage-grouse) and *Lagopus leucura* (white-tailed ptarmigan), have become pseudogenes. Pseudogenes of these two grouses share two frame-shifts, suggesting a common origin. Five paleognath species we surveyed also seem to have lost the functional pinopsin gene.While we found pinopsin pseudogenes in the kiwi, *Apteryx rowi*, and the common ostrich, *Struthio camelus*, we failed to find any in the emu, *Dromaius novaehollandiae*, the Chilean tinamou, *Nothoprocta perdicaria*, or the white-throated tinamou, *Tinamus guttatus* using TBLASTN searches (Evalue < 0.05). There is a genome-assembly gap in the upstream region of the ostrich pinopsin pseudogene. GenBank/DDBJ/EMBL accession IDs of predicted proteins are provided in Supplementary Table S5. shift = frame-shift.

**Supplementary Figure S6**

A molecular phylogenetic tree of parapinopsin, parapinopsin-like, and QB-opsin, as well as representative members of other subfamilies generated by the BI method, based on alignment of GPCR domains. Parapinopsin and parapinopsin-like make a single clade, and QB opsin is placed outside these two. Lampreys have three parapinopsin-like paralogs. The ML method yielded a similar result. Posterior probabilities of BI and bootstrap values of ML are shown in this order at major nodes. The amino acid at position 113 is shown on the right side of each opsin subfamily name. For amino acids at position 113 of subfamilies other than QB opsin and parapinopsin-like, see Lamb (2013).

Lamb TD. 2013. Evolution of phototransduction, vertebrate photoreceptors and retina. Prog Retin Eye Res. 36:52-119.

**Supplementary Figure S7**

Molecular phylogenetic analysis by BI of (A) NT5DC2 and related sequences (B) TNNC1 and related sequences used in the microsynteny analysis shown in Fig. 4. ML yielded similar results. Posterior probabilities of BI and bootstrap values of ML are shown in this order at major nodes. Predicted proteins shown in Fig. 4 are highlighted in yellow.

**Supplementary Figure S8**

Microsynteny around Parapinopsin loci is highly conserved, although in many species, this opsin is missing. Protein coding genes are shown. Distances between these segments are based on TBLASTN search results (Supplementary Fig. S9). H = ERC2, I = WNT5A, J = CACNA2D3, K = SELENOK, L = ACTR8, M = IL17RB, and N = CHDH. GenBank/DDBJ/EMBL accession IDs of H to N of these species are shown in Supplementary Table S5.

**Supplementary Figure S9**

Sequence alignments from TBLASTN searches for QB opsin pseudogenes against the refseq_genome database of each species. Fence lizard QB opsin (XP_042303413) was used as a query. Evalue < 0.05. Matrix = BLOSUM45. Regions used for molecular phylogenetic analyses in Supplementary Figure S10 are underlined.

**Supplementary Figure S10**

Pseudogenized nucleotide sequences of QB opsin of the coelacanth, the sterlet, and the spotted gar make a statistically significant single clade with functional QB opsins. (A) Regions corresponding to a part of the QB opsin second exon and (B) those of the third exon were used. First, translated sequences of QB opsin pseudogenes yielded by TBLASTN (Supplementary Figure S9 for details) and functional opsin amino acid sequences were aligned using MAFFT (Katoh et al. 2002; version 7.245). These alignments were improved manually. Then, corresponding nucleotide alignments were subjected to molecular phylogenetic analyses by ML using PhyML 3.0 (Guindon et al. 2010) with 100 bootstrap pseudoreplications with the GTR substitution model. Numbers at nodes indicate bootstrap values. Rooted trees are shown for simplicity. Molecular phylogenetic trees and alignments are available on FigShare (doi: 10.6084/m9.figshare.28300961).

Guindon S, Dufayard JF, Lefort V, Anisimova M, Hordijk W, Gascuel O. 2010. New algorithms and methods to estimate maximum-likelihood phylogenies: assessing the performance of PhyML 3.0. Syst Biol. 59:307-321.

Katoh K, Misawa K, Kuma K, Miyata T. 2002. MAFFT: a novel method for rapid multiple sequence alignment based on fast Fourier transform. Nucleic Acids Res. 30:3059-3066.

**Supplementary Figure S11**

A scenario of how QB opsin, parapinopsin, and parapinopsin-like, as well as other opsin subfamilies of VVNVO, may have emerged in the course of evolution. We propose that (1) parapinopsin and parapinopsin-like were duplicated by 1R (2) (parapinopsin + parapinopsin-like) and QB opsin were produced by a local tandem duplication event that preceded WGDs. We also propose that three parapinopsin-like paralogs of lampreys emerged by 2RCY (indicated with an asterisk). Local gene duplication events are shown with open squares, and gene duplications by 1R with open circles.
